# Supplementary material for: Cytotoxic Vδ2+ T cell subsets expand in response to malaria in human tonsil and spleen organoids
Source: PLoS Pathog. 2026 Apr 10;22(4):e1013565. doi: 10.1371/journal.ppat.1013565 (PMC13102301; doi:10.1371/journal.ppat.1013565)
Supplement: S1 Fig — Microscope image showing formation of germinal centers in tonsil organoid stimulated with iRBC. B. Cell frequencies (of all single cells) at day 21/28 (1–3 donors depending on cell type). C. Gating strategy for B cell panel. D. Gating strategy for T cell panel. E. Gating strategy for innate cell panel. F. Frequencies of cells that do not change in tonsil organoids in response to iRBC (compared to uRBC). N = 10. Bars indicate medians. P-values were calculated using the Wilcoxon ranked sum test with correction for multiple comparisons using Benjamini-Hochberg method (FDR 0.05). G. Expression of HLA-DR on Vδ2 + T cells from 14 tonsil donors with both D7 and D14 timepoints, and 3 additional donors with only D7. Bars indicate medians. H. Tonsil organoids from 3 donors were stimulated for 14 days and a select set of Vδ2 + T cell surface markers were evaluated. Frequencies of Vδ2 + T cell subsets expressing Tim-3 and CD8, and frequencies or of central memory and effector memory subsets were assessed at multiple timepoints and conditions. Bars indicate medians. (DOCX) [file ppat.1013565.s002.docx]

*
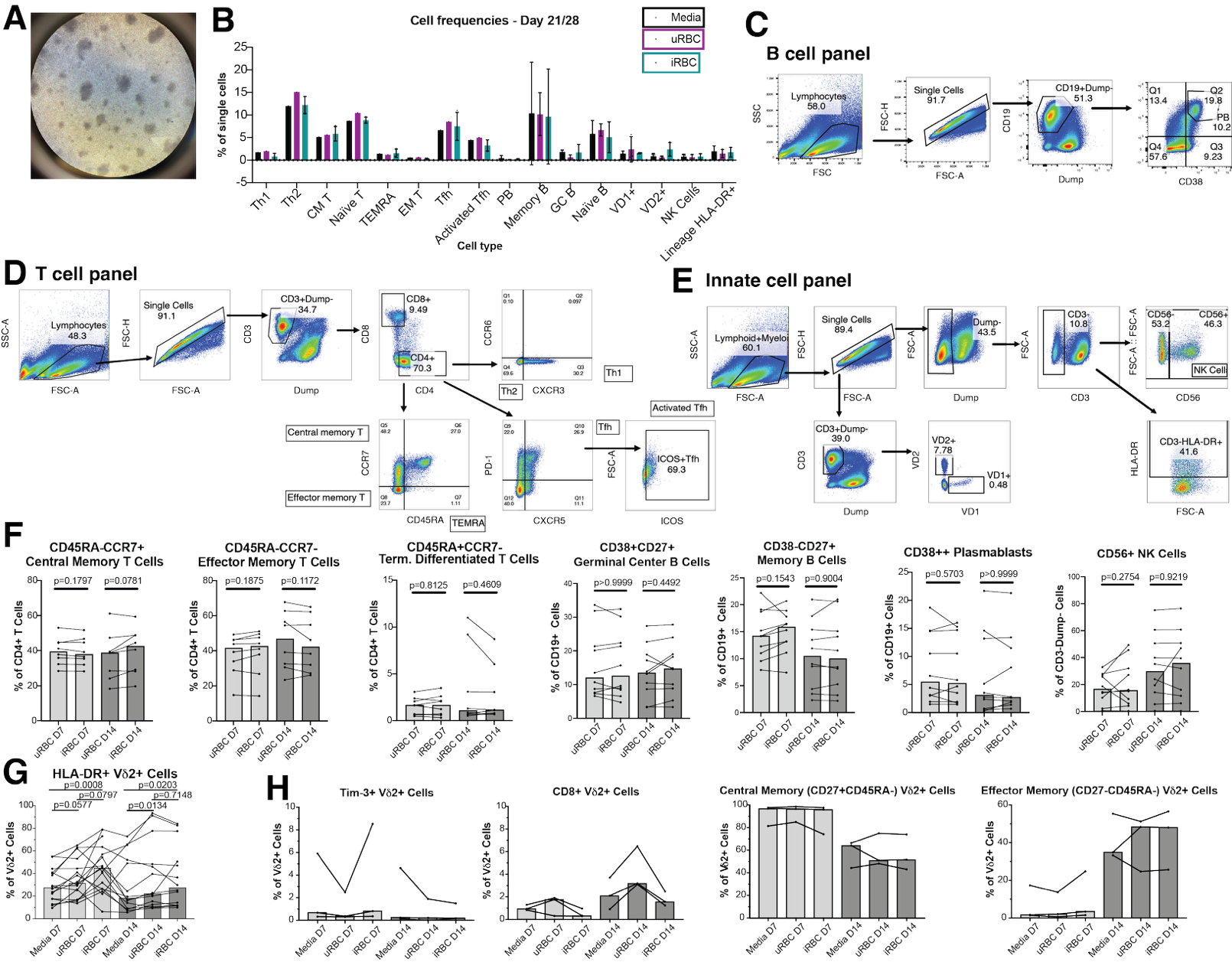
***S1 Fig**

*S1 Fig: Germinal centers form in organoids in response to iRBC stimulation*

A. Microscope image showing formation of germinal centers in tonsil organoid stimulated with iRBC.

B. Cell frequencies (of all single cells) at day 21/28 (1-3 donors depending on cell type).

C. Gating strategy for B cell panel.

D. Gating strategy for T cell panel.

E. Gating strategy for innate cell panel.

F. Frequencies of cells that do not change in tonsil organoids in response to iRBC (compared to uRBC). N=10. Bars indicate medians. P-values were calculated using the Wilcoxon ranked sum test with correction for multiple comparisons using Benjamini-Hochberg method (FDR 0.05)

G. Expression of HLA-DR on Vδ2+ T cells from 14 tonsil donors with both D7 and D14 timepoints, and 3 additional donors with only D7. Bars indicate medians.

H. Tonsil organoids from 3 donors were stimulated for 14 days and a select set of Vδ2+ T cell surface markers were evaluated. Frequencies of Vδ2+ T cell subsets expressing Tim-3 and CD8, and frequencies or of central memory and effector memory subsets were assessed at multiple timepoints and conditions. Bars indicate medians.
