# Supplementary material for: Cytotoxic Vδ2+ T cell subsets expand in response to malaria in human tonsil and spleen organoids
Source: PLoS Pathog. 2026 Apr 10;22(4):e1013565. doi: 10.1371/journal.ppat.1013565 (PMC13102301; doi:10.1371/journal.ppat.1013565)
Supplement: S2 Fig — A. Expression of markers defining all cell clusters. Numbers correspond to clusters in Fig 2B. B. Expression of antibody surface markers across all clusters. C. Expression of TCR gamma chain genes (left) and delta chain genes (right) across cell clusters. D. Expression of class II antigen presentation markers across clusters. E. UMAP showing distribution of select genes associated with cell division that are specifically enriched in Vδ2 + cells after iRBC stimulation. See Fig 2B for the location of γδ T cell clusters. (DOCX) [file ppat.1013565.s003.docx]

**
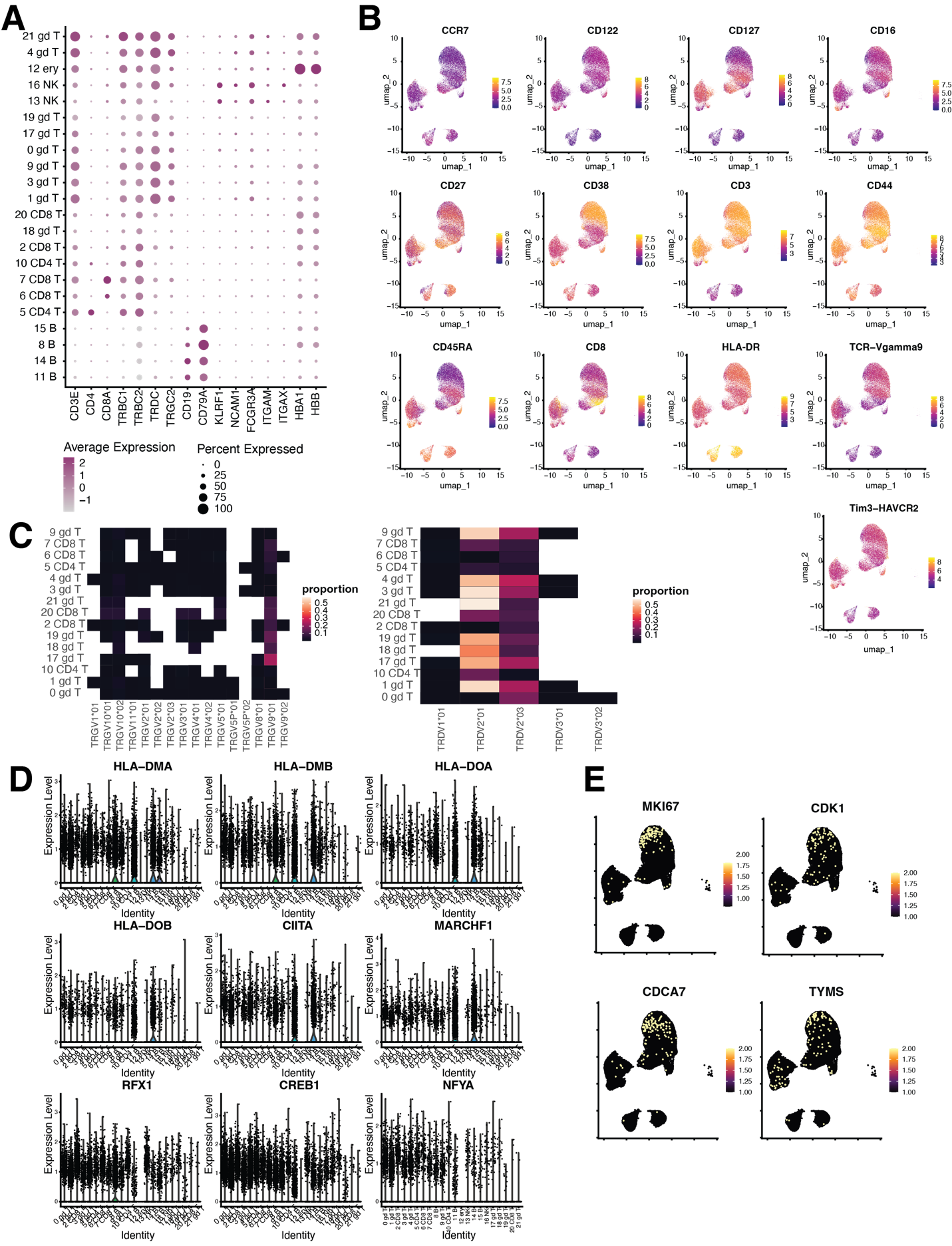
S2 Fig**

*S2 Fig: AbSeq and RNA expression defines cell clusters*

A. Expression of markers defining all cell clusters. Numbers correspond to clusters in Fig 2B.

B. Expression of antibody surface markers across all clusters.

C. Expression of TCR gamma chain genes (left) and delta chain genes (right) across cell clusters.

D. Expression of class II antigen presentation markers across clusters

E. UMAP showing distribution of select genes associated with cell division that are specifically enriched in Vδ2+ cells after iRBC stimulation. See Fig 2B for the location of γδ T cell clusters.
