# Supplementary material for: Cytotoxic Vδ2+ T cell subsets expand in response to malaria in human tonsil and spleen organoids
Source: PLoS Pathog. 2026 Apr 10;22(4):e1013565. doi: 10.1371/journal.ppat.1013565 (PMC13102301; doi:10.1371/journal.ppat.1013565)
Supplement: S3 Fig — A. Bar graph showing the percentage of each donor’s conventional T cell receptor repertoire occupied by clones of different orders of magnitude. B. Alluvial plot showing the distribution of the 10 largest conventional T cell clones per sample. Only cells with a high confidence α and β chain were included. (DOCX) [file ppat.1013565.s004.docx]

**S3 Fig**
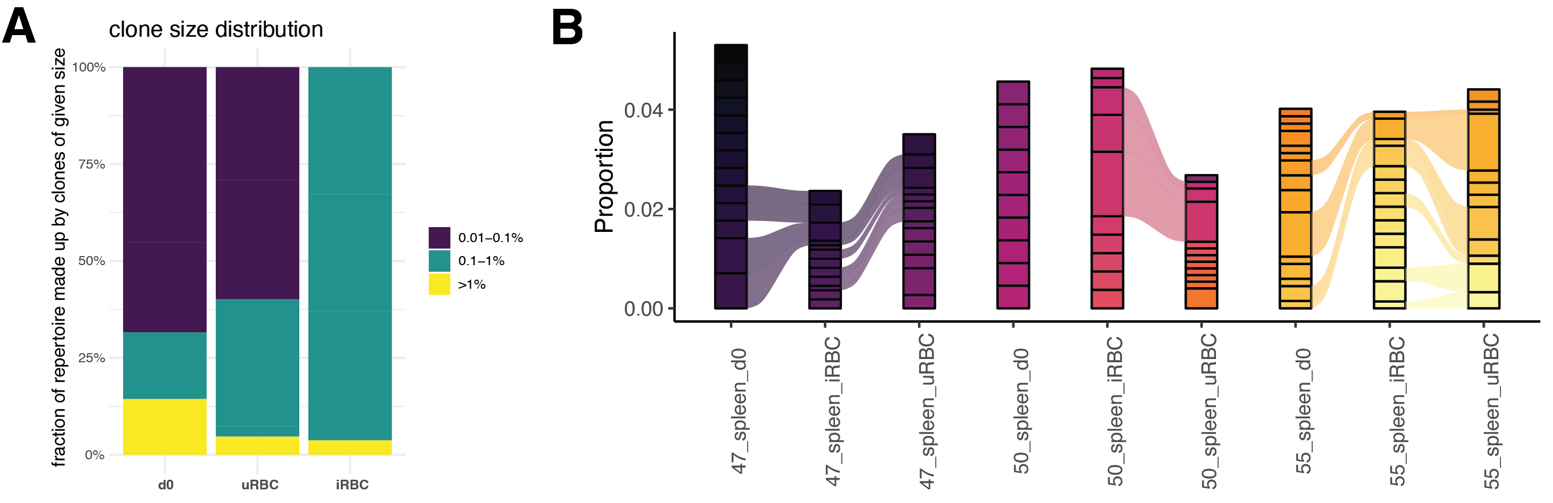


*S3 Fig: Receptor repertoires of conventional T cells in the spleen organoid are relatively stable between experimental conditions.*

A. Bar graph showing the percentage of each donor’s conventional T cell receptor repertoire occupied by clones of different orders of magnitude.
B. Alluvial plot showing the distribution of the 10 largest conventional T cell clones per sample. Only cells with a high confidence α and β chain were included.
