# Supplementary material for: Cytotoxic Vδ2+ T cell subsets expand in response to malaria in human tonsil and spleen organoids
Source: PLoS Pathog. 2026 Apr 10;22(4):e1013565. doi: 10.1371/journal.ppat.1013565 (PMC13102301; doi:10.1371/journal.ppat.1013565)
Supplement: S4 Fig — A. Vδ2- frequency of total T cells. B. Frequency of Vδ2- T effector memory (TEM: CD27-CD45RA-), T central memory (TCM: CD27 + CD45RA-), T terminally differentiated effector memory (TEMRA: CD27-CD45RA+) and T naive (TNAIVE: CD27 + CD45RA+). C. Frequency of Vδ2- T cell intracellular Granzyme-B expression. Comparisons performed by Mann-Whitney U test. Center line representing the median, box limits indicating the upper and lower quartiles, whiskers extending to 1.5 times the interquartile range. D. Pie chart depicts co-expression of CD38, CD107a, Granzyme-B, IFNγ and TNF by Vδ2- T cells. Comparisons performed by permutation test. E. Frequency of CD38+CD107a+Granzyme-B+IFNγ+TNF+ and CD38+CD107a+Granzyme-B-IFNγ+TNF+ Vδ2- T cells. Bar representing mean and whiskers extend to standard deviation. Comparisons performed by Mann-Whitney U test. (DOCX) [file ppat.1013565.s005.docx]

**S4 Fig**


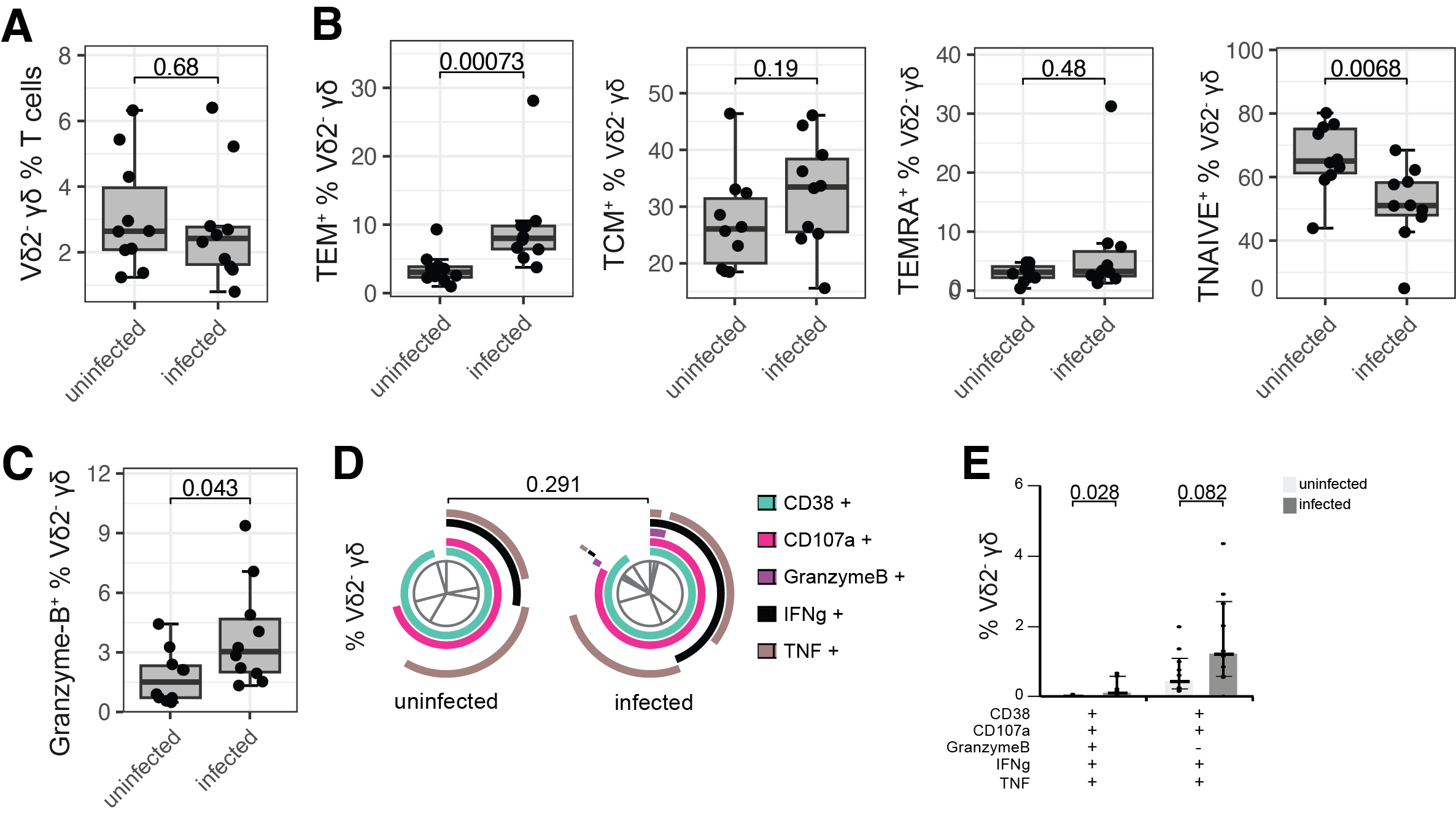


*S4 Fig: Changes in Vδ2- T cell phenotypes in malaria infected children* Isolated Tonsillar mononuclear cells from malaria-endemic Ugandan children with an asymptomatic malaria infection (infected) or no malaria infection (uninfected) were stimulated with PMA and Ionomycin for 24 hours.

A. Vδ2- frequency of total T cells.

B. Frequency of Vδ2- T effector memory (TEM: CD27-CD45RA-), T central memory (TCM: CD27+CD45RA-), T terminally differentiated effector memory (TEMRA: CD27-CD45RA+) and T *naive* (TNAIVE: CD27+CD45RA+).

C. Frequency of Vδ2- T cell intracellular Granzyme-B expression.

Comparisons performed by Mann-Whitney U test. Center line representing the median, box limits indicating the upper and lower quartiles, whiskers extending to 1.5 times the interquartile range.

D. Pie chart depicts co-expression of CD38, CD107a, Granzyme-B, IFNγ and TNF by Vδ2- T cells. Comparisons performed by permutation test.

E. Frequency of CD38^+^CD107a^+^Granzyme-B^+^IFNγ^+^TNF^+^ and CD38^+^CD107a^+^Granzyme-B^-^IFNγ^+^TNF^+^ Vδ2- T cells. Bar representing mean and whiskers extend to standard deviation. Comparisons performed by Mann-Whitney U test.
