# Supplementary material for: Cytotoxic Vδ2+ T cell subsets expand in response to malaria in human tonsil and spleen organoids
Source: PLoS Pathog. 2026 Apr 10;22(4):e1013565. doi: 10.1371/journal.ppat.1013565 (PMC13102301; doi:10.1371/journal.ppat.1013565)
Supplement: S5 Fig — T cell subsets, B cell subsets, and most innate cell frequencies, whether of all single cells (A) or of parent gate (B), are not different following stimulation with iRBC + LAIV compared to the uRBC + LAIV or LAIV alone conditions. LAIV was added at day 0. N = 3. C. Vδ2 + T cell frequencies are significantly different between iRBC+LAIV and uRBC+LAIV or LAIV alone at Dat 7 and Day 14. N = 6. D. Vδ2 + T cell frequencies do not change following LAIV stimulation compared to media controls (N = 6, 3 for LAIVd0, 3 for LAIVd3). (DOCX) [file ppat.1013565.s006.docx]

**S5 Fig**
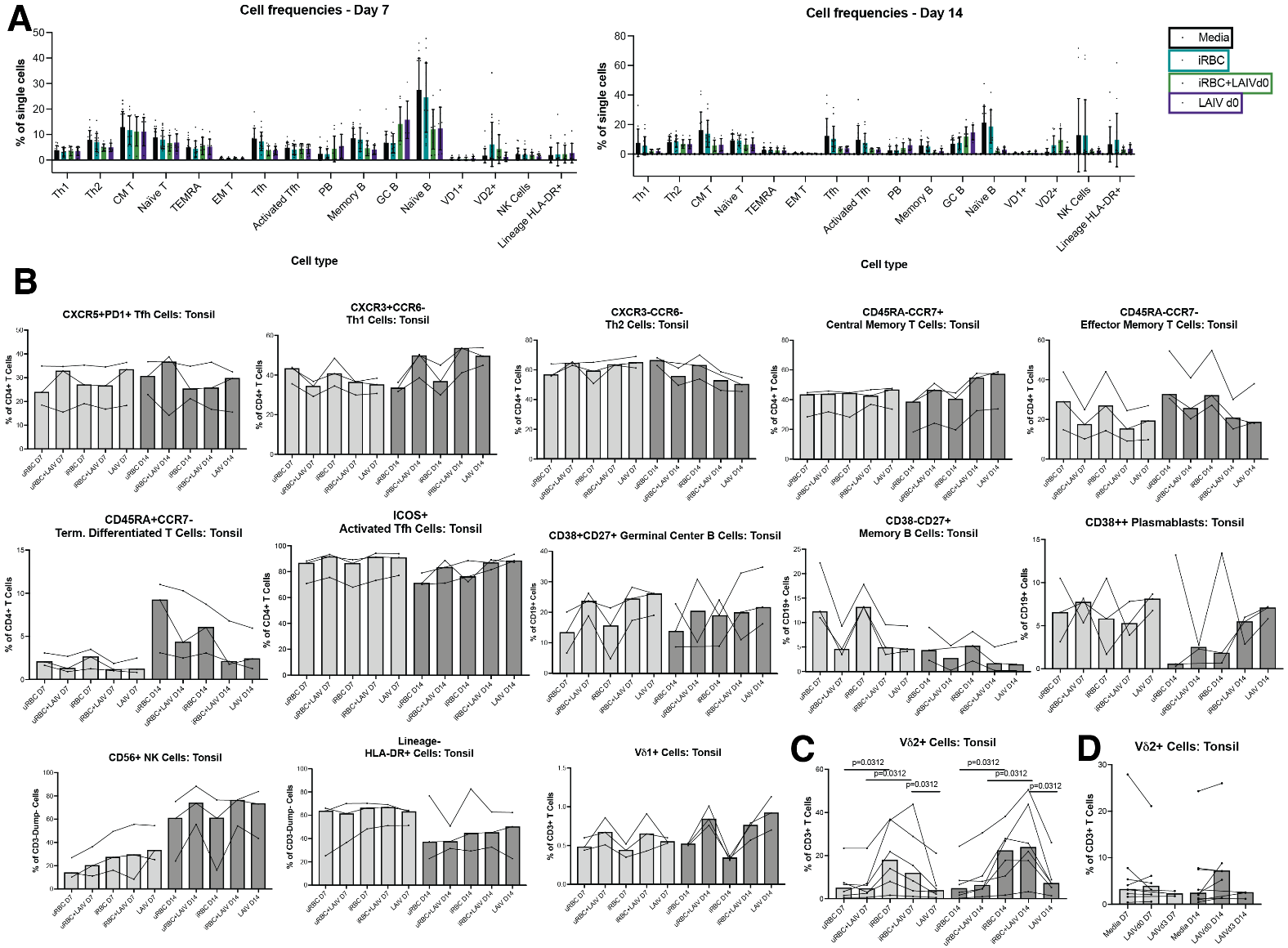


*S5 Fig:* *iRBC exposure does not impact cellular frequencies following LAIV vaccine*

T cell subsets, B cell subsets, and most innate cell frequencies, whether of all single cells (A) or of parent gate (B), are not different following stimulation with iRBC + LAIV compared to the uRBC + LAIV or LAIV alone conditions. LAIV was added at day 0. N=3. C. Vδ2+ T cell frequencies are significantly different between iRBC+LAIV and uRBC+LAIV or LAIV alone at Dat 7 and Day 14. N=6. D. Vδ2+ T cell frequencies do not change following LAIV stimulation compared to media controls (N=6, 3 for LAIVd0, 3 for LAIVd3).
