# Supplementary material for: Cytotoxic Vδ2+ T cell subsets expand in response to malaria in human tonsil and spleen organoids
Source: PLoS Pathog. 2026 Apr 10;22(4):e1013565. doi: 10.1371/journal.ppat.1013565 (PMC13102301; doi:10.1371/journal.ppat.1013565)
Supplement: S6 Fig — A. Antibody responses to flu protein from ELISAs using supernatants from cultures exposed to different stimulation conditions at Day 7, Day 14, or Day 21/28. Lines represent unique donors. Tonsil donors are indicated in black and spleens in red. Area under the curve (AUC) was calculated using the curve of absorbances across the 4 dilutions tested (neat, 1:10, 1:100, 1:300). B. Example curve of absorbances across 4 dilutions that was used to calculate AUC. C. IgG antibody response to malaria schizont extract lysate from ELISAs using undiluted supernatants from organoid cultures exposed to different stimulation conditions. Ugandan plasma was added at 1:1000. D. IgG antibody response to malaria parasite antigens (MSP1, AMA1, MSP2) from ELISAs using supernatants (1:10 or 1:100) from organoid cultures exposed to different stimulation conditions. Ugandan plasma was added at 1:1000.E. IgM antibody response to malaria schizont extract lysate from ELISAs using supernatants from unstimulated, uRBC-stimulated or iRBC-stimulated cultures at day 7 (n = 13 tonsils and 1 spleen). Area under the curve (AUC) was calculated using the curve of absorbances across the 4 dilutions tested (neat, 1:10, 1:100, 1:300).F. IgM antibody response to malaria schizont extract lysate from ELISAs using undiluted supernatants from unstimulated vs. iRBC-stimulated organoid cultures (n = 14 tonsils and 3 spleens) at days 7 and 14. Absorbance values are average of 2 replicates. (DOCX) [file ppat.1013565.s007.docx]

**S6 Fig**

*
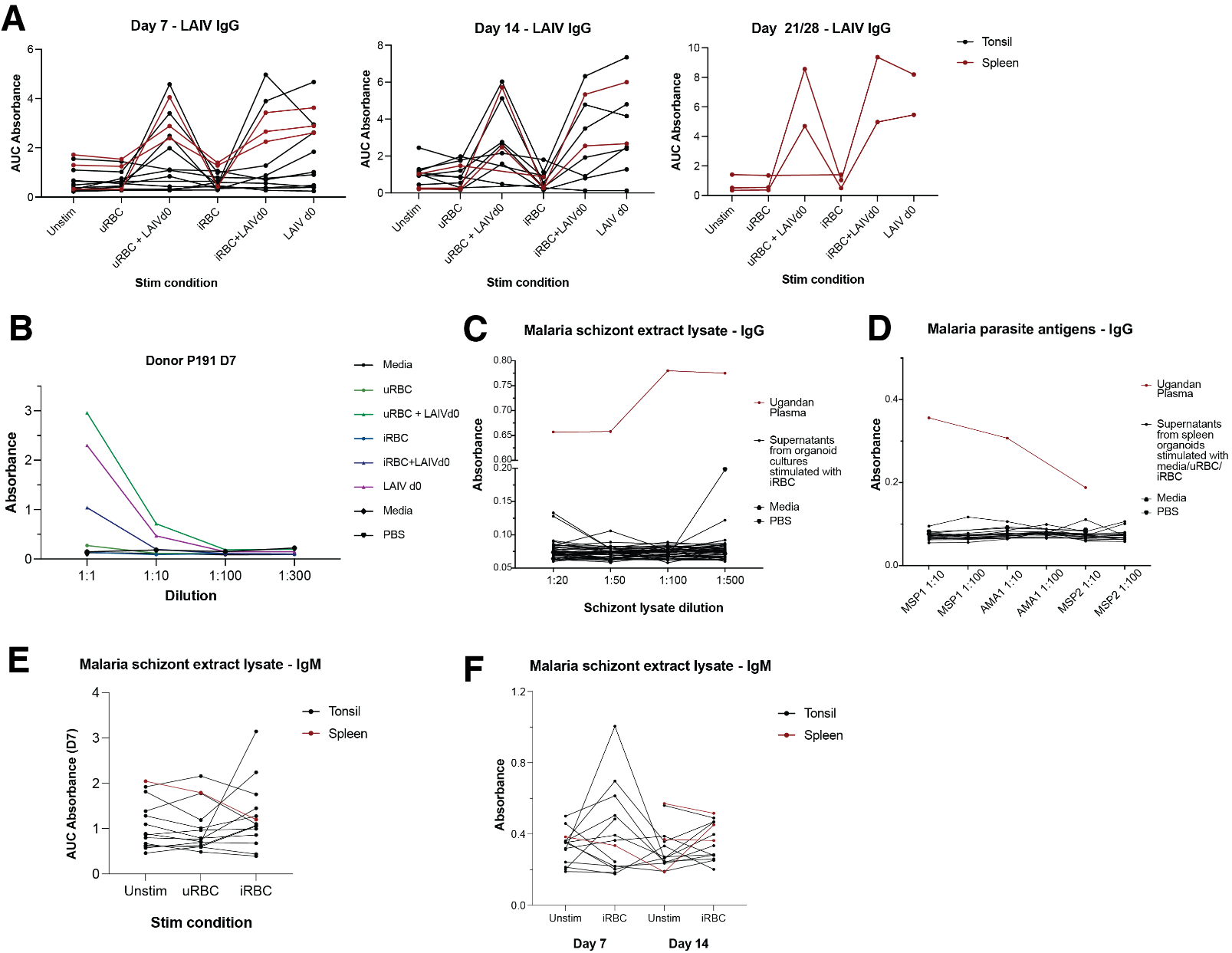
S6 Fig:* *iRBC impacts antibody response to LAIV vaccine but not malaria parasites*

A. Antibody responses to flu protein from ELISAs using supernatants from cultures exposed to different stimulation conditions at Day 7, Day 14, or Day 21/28. Lines represent unique donors. Tonsil donors are indicated in black and spleens in red. Area under the curve (AUC) was calculated using the curve of absorbances across the 4 dilutions tested (neat, 1:10, 1:100, 1:300).

B. Example curve of absorbances across 4 dilutions that was used to calculate AUC.

C. IgG antibody response to malaria schizont extract lysate from ELISAs using undiluted supernatants from organoid cultures exposed to different stimulation conditions. Ugandan plasma was added at 1:1000.

D. IgG antibody response to malaria parasite antigens (MSP1, AMA1, MSP2) from ELISAs using supernatants (1:10 or 1:100) from organoid cultures exposed to different stimulation conditions. Ugandan plasma was added at 1:1000.

E. IgM antibody response to malaria schizont extract lysate from ELISAs using supernatants from unstimulated, uRBC-stimulated or iRBC-stimulated cultures at day 7 (n=13 tonsils and 1 spleen). Area under the curve (AUC) was calculated using the curve of absorbances across the 4 dilutions tested (neat, 1:10, 1:100, 1:300).

F. IgM antibody response to malaria schizont extract lysate from ELISAs using undiluted supernatants from unstimulated vs. iRBC-stimulated organoid cultures (n=14 tonsils and 3 spleens) at days 7 and 14. Absorbance values are average of 2 replicates.
