# Supplementary material for: Cytotoxic Vδ2+ T cell subsets expand in response to malaria in human tonsil and spleen organoids
Source: PLoS Pathog. 2026 Apr 10;22(4):e1013565. doi: 10.1371/journal.ppat.1013565 (PMC13102301; doi:10.1371/journal.ppat.1013565)
Supplement: S7 Fig — Experiments were performed in which γδ + T cells were depleted from organoids prior to 7-day stimulation with uRBC/iRBC + /- LAIV. Effectiveness of Vδ2+ and Vδ1 + depletion is shown in A. T cell responses (B), B cell responses C), and innate cell responses (D) are no different between conditions containing γδ + T cells or depleted of γδ + T cells. (DOCX) [file ppat.1013565.s008.docx]

**S7 Fig**


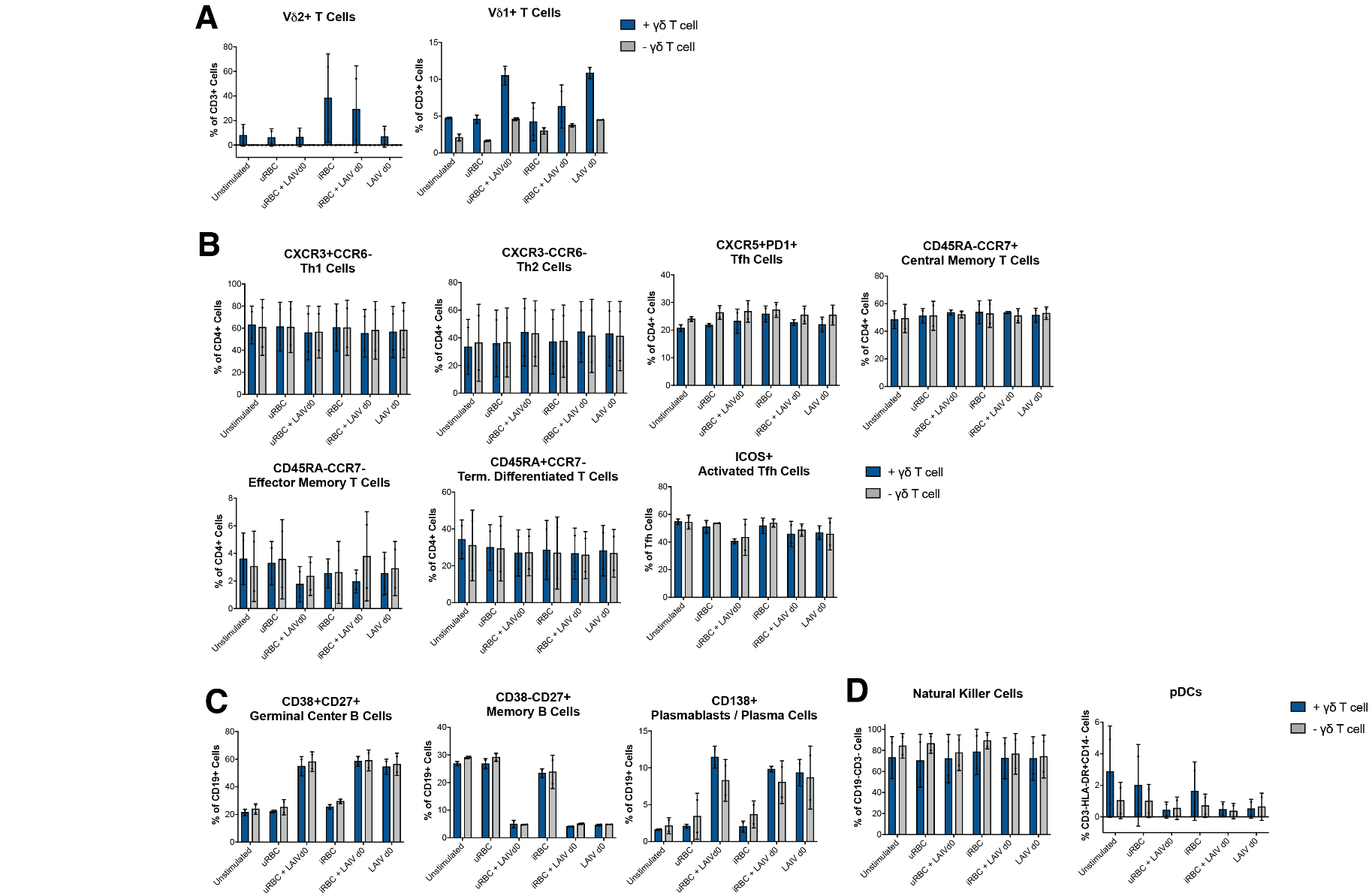
*S7 Fig:* *γδ+ T cell depletion does not impact immune responses at day 7*

Experiments were performed in which γδ+ T cells were depleted from organoids prior to 7-day stimulation with uRBC/iRBC +/- LAIV. Effectiveness of Vδ2+ and Vδ1+ depletion is shown in A. T cell responses (B), B cell responses C), and innate cell responses (D) are no different between conditions containing γδ+ T cells or depleted of γδ+ T cells.
