# Supplementary material for: Cytotoxic Vδ2+ T cell subsets expand in response to malaria in human tonsil and spleen organoids
Source: PLoS Pathog. 2026 Apr 10;22(4):e1013565. doi: 10.1371/journal.ppat.1013565 (PMC13102301; doi:10.1371/journal.ppat.1013565)
Supplement: Attachment — Submitted filename: Response to Reviewers.docx [file ppat.1013565.s011.docx]

**Reviewers' Comments:**

Reviewer's Responses to Questions

**Part I - Summary**
Please use this section to discuss strengths/weaknesses of study, novelty/significance, general execution and scholarship.

Reviewer #1: This manuscript by Press et al, uses human tonsil and splenic organoids to examine the response to Plasmodium infected red blood cells (iRBCs) and to live attenuated influenza vaccine. After incubation over 14-28 days (depending on organoid) the major change in cell populations was the expansion of Vd2 GD T cells. This is a small population in the culture but does appear to be the only real difference observed.

The novelty of this study is that it is the first to examine responses during Plasmodium infection using tonsil / splenic cultures. I appreciate that this work is not trivial to carry out. The analysis of the GD T cells are insightful and demonstrate how organoid cultures could be used to dissect some of the responses observed in human studies. As such I can see this study would be helpful to the community in some regards. However, unfortunately I am not sure that the data supports a general utility for this approach with the current optimization / data obtained. If gamma delta cells are the primary difference how would one go about studying the other cells of the immune system with this approach? It perhaps needs more optimization.

We thank the reviewer for their comments and appreciate their understanding of the complexity of experiments in human organoids. The tonsil and spleen organoid systems have been extensively optimized by the Davis lab, who have observed changes in numerous T and B cell subsets in response to influenza (Wagar et al. 2021). In adapting the system to malaria, we tested a wide range of iRBC doses and lengths of culture. We agree that in the future, further optimization of the iRBC-specific system would be beneficial (see discussion, lines 443-449).

While we focused on Vδ2+ γδ T cells since they showed the strongest responses to iRBC at multiple timepoints, we did see changes in other cell types, including activated Tfh at day 14 (Fig 1D) and HLA-DR+ innate cells at day 7 (Fig 1E). Other cells (e.g. Th1, Th2, Tfh, Vδ1+ T cells) do show differences, but these differences were not significant following multiple comparisons correction (this was noted during the revision, lines 154-157)). This has now been clarified by adding asterisks in Figure 1 for the p-values that are statistically significant following multiple comparisons correction. These trends, however, do suggest that there could be additional changes in other cell types that are obscured by donor variability and that could be elucidated by future experiments using more donors. This has now been included in the discussion (lines 412-414).

Additionally, while numerous immune cells are of course important in responses to malaria, recent malaria vaccine studies have also identified Vδ2+ γδ T cells as the cell type most associated with protection from malaria (see Introduction). Together, our results are consistent with other studies and support use of the organoid system to study diverse immune cells in response to multiple pathogens.

Reviewer #2: This study by Press et al seeks to explore lymphoid tissue responses of Vdelta2 T cells (Vd2+) in tonsil and spleen organoids. They look at cellular responses of Vd2+ cells in spleen organoids in vitro and tonsils from donors from Uganda. The main findings indicate Vd2+ cells expand in organoid cultures and this is underpinned by transcriptional signatures of cytolytic effector cells and clonal expansion of the gd TCR.

Reviewer #3: Strengths of the Study:
This ex vivo model represents a relatively new and emerging tool of the lymphoid organoid system for studying malaria immunology and vaccine efficacy.

Press et al. provide a valuable human lymphoid-organoid model for studying malaria immune responses, in particular, understanding γδ T-cell biology in human lymphoid tissues during primary malaria infection.

Weaknesses / Major Concerns:

The authors should discuss their findings in greater depth and align their conclusions with the existing literature comparing non-endemic adults with endemic adults and children. The authors should clearly state that the organoid model primarily reflects a first malaria infection and cannot be extrapolated to repeated infections.

We appreciate this suggestion and agree that clearly distinguishing between responses in naïve/non-endemic vs. chronic/endemic populations are important. We have made several changes to clarify this:

- edited the introduction (lines 86-95) to highlight these differences
- clarified in lines 161-163 and 384-385 that our observed proliferation of Vδ2+ γδ T cells is consistent with previous work in **malaria-naïve** individuals
- added the sentence “The organoid model represents an acute malaria infection while most individuals in malaria-endemic countries experience chronic infections” as an introduction to the experiments using Ugandan tonsils (lines 289-290)
- mentioned that our observed lack of Vδ1+ T cell expansion “could be explained by the organoid representing a primary infection rather than a setting of chronic infection” (lines 430-431)

Given that we observed an increase in activation and cytotoxic markers in both naïve and Ugandan tonsils, we think the clonal expansion of cytotoxic Vδ2+ subsets could be similar in primary and secondary infections; however, this would of course need to be further studied with more donors and could also be dependent on number of previous infections experienced.

The interpretation of the Uganda children’s tonsils requires caution, since Vδ2⁺ frequencies were not increased in infected children (Fig. 4A), in contrast to the organoid cultures. Moreover, studies analysing human peripheral blood consistently report a decrease in Vδ2 T cells in chronically exposed populations (Jagannathan et al., 2014; Diallo et al., 2019). The authors should interpret the tonsillar cytotoxic phenotype in this context and extrapolate with caution and rigor to the γδ T-cell analyses in individuals from endemic regions.

Thank you for this point. As the reviewer notes above, there are absolutely differences in responses between endemic and non-endemic populations, and results in endemic populations are often less clear and likely related to previous history of malaria. For example, Harawa et al. (2019) observed decreased frequencies during an active infection (whether severe or asymptomatic) in Malawian children while Diallo et al. (2019) observed the opposite in Malian children. Jagannathan et al. (2014) notesdno change in Vδ2⁺ frequencies during an active infection in Ugandan children chronically exposed to malaria (despite a reduced capacity to proliferate following repeated infections), which is consistent with our observed lack of Vδ2⁺ expansion in Ugandan tonsils.

Despite differing conclusions about expansion of total Vδ2⁺ (or Vδ2-) T cells, numerous studies do support an increase in expression of activation markers in infected individuals in endemic regions – we have added these references in lines 170 and 412. These results may appear contradictory with other reports of reduced pro-inflammatory markers and increased immunoregulatory markers among children exposed to repeated malaria (Jagannathan et al., 2014; Farrington et al., 2016; Jagannathan et al., 2017; Schofield et al. 2017). However, Vδ2+ T cell subsets are complex and could exert multiple functions or different subsets could expand at different timepoints following infection (added to Discussion in lines 405-409).

The authors should also assess Vδ1 and Vδ3 γδ T-cell subsets in the Ugandan children's tonsils. These subsets are predominant in human lymphoid tissues, such as pediatric tonsils, and often expand or differentiate into effector cells during malaria infection. It is important to report, if possible, the Vδ1/Vδ3 potential contribution to the CD27⁺CD45RA⁺ T-naïve γδ population, as well as the frequencies and activation states in these children. Including these data would substantially strengthen the manuscript by providing a more complete picture of γδ T-cell repertoire in the lymphoid tissues of malaria-exposed children.

Thank you for this valuable suggestion. We have added data on Vδ2- γδ T cells in Ugandan donors to Figure S4 and lines 310-313. Among Ugandan donors, a higher percentage of γδ T cells were Vδ2- as compared to Vδ2+, and Vδ2- γδ T cells had similar differences in TEM and Granzyme B between uninfected and infected donors. We did also look for Vδ1 genes in our single-cell experiment clusters, but were unable to identify any alignment indicating presence of Vδ1+or Vδ3+ T cells (added to Figure S2C). We hypothesize that this could be explained by the donors being malaria-naïve or by a difference in Vδ2+ and Vδ2- γδ T cells in their capacity to expand over 2 weeks in culture.

The authors claim that Vδ2⁺ activation is malaria-antigen specific by showing the expression of activation markers. However, without specific mechanistic assays (Sandstrom et al., Science, 2014; Schofield et al., BMC Med, 2017), the conclusion is indirect and should be stated more cautiously.

We believe that our data supports malaria-specific responses but agree that this is not definitive and additional mechanistic assays are needed to support this claim. We have removed the term “antigen-specific” from the Abstract. In line 62, we state that the single cell analysis “suggests antigen-specific responses”. In line 174, we add that the increase in Vδ2+ T cell abundance in the iRBC condition “strongly suggests” that Vδ2+ T cells respond to malaria. We additionally added the sentence “Mechanistic assays (using cells from both naïve and malaria-exposed individuals) could confirm that the observed Vδ2+ T cell activation is malaria specific” in lines 410-412.

In the study, Vγ9Vδ2 T cells upregulated surface expression of APC markers, HLA-DR, CD74, TAP, and CIITA, in response to iRBCs. However, this expression is insufficient to attribute functional antigen processing and presentation (Brandes et al., Science, 2005; Howard et al, 2017). The authors should revise this by stating that this conclusion requires experimental validation.

We agree protein or RNA expression of antigen presentation markers, while supporting that antigen presentation could occur, is insufficient to conclusively show that it does occur. We feel that our language is consistent with this, as we state:

- “This upregulation of antigen presentation genes is consistent with our observation that HLA-DR surface expression increased following iRBC stimulation (Fig S1G), **suggesting** that Vδ2+ T cells may not simply be responding to P. falciparum antigen, but **may** present antigen as well.” (lines 224-225)
- “the observed upregulation of class I and class II antigen-processing genes in clusters enriched following iRBC stimulation **warrants further investigation** into Vδ2+ T cells as **potential** antigen-presenting cells” (lines 408-409)

However, we would be happy to edit these statements if the reviewers feel they are misrepresenting the potential for antigen presentation.

**Part II – Major Issues: Key Experiments Required for Acceptance**
Please use this section to detail the key new experiments or modifications of existing experiments that should be absolutely required to validate study conclusions.

Generally, there should be no more than 3 such required experiments or major modifications for a "Major Revision" recommendation. If more than 3 experiments are necessary to validate the study conclusions, then you are encouraged to recommend "Reject".

Reviewer #1: Required experiments: I think the data are surprising. For example, I would have expected more changes than just GD T cells. There are very few other changes observed. This makes me curious as to the applicability of the organoid system given the length of time cells were cultured. NK cells are another innate cell where there does not appear significant changes despite published literature demonstrating both responsiveness to Plasmodium iRBCs and importance in animal models. One wonders whether perhaps there is some issue with the infusion of Plasmodium iRBCs / LAIV that may need adjustment? One thing that springs to mind is that perhaps the authors should consider adding purified merozoites – whilst schizonts are immunogenic, there are several reports that point to the immunogenicity of the merozoites contained inside. Also hemozoin may be interfering with responses. What do the organoids do if merozoites are added instead of iRBCs?

As mentioned above, we did see changes in some T cell and innate cell subsets in response to iRBC and Wagar et al. saw responses in numerous B and T cell subsets in response to influenza. NK cells are a very small population in the tonsil (DOI: [10.1016/j.jaci.2024.11.018](https://urldefense.com/v3/__https:/doi.org/10.1016/j.jaci.2024.11.018__;!!G5ONXFxL7-lpCg!aM_XJc7vaxPhyk6n9e0kGy2SczLRFoUR-VP2rI2RftYqdmRu_CAM2wkAV3rFHb7LkJ1U2Shwmsit5DMPmUGyR8si7Jw0Lb9gbQ$)), so we would not expect to see major changes in NK cells in organoids despite their significance in human and animal malaria.

Studies have shown that all parasite life cycles, including the intact infected RBC are found in lymphoid tissues (particularly spleens—Kho et al., DOI: [10.1056/NEJMc2023884)](https://www.nejm.org/doi/full/10.1056/NEJMc2023884) during malaria. As such we used iRBCs which have antigens from both the surface and merozoite stages. We agree that individual parasite stages or parasite components may down regulate immune responses and it is this down regulation that we were testing when restimulating with influenza. For hemozoin in particular, there is evidence that hemozoin impairs dendritic cell function in mice (DOI: 10.1186/jbiol34; DOI: 10.1371/journal.ppat.0030143); however, this has not been supported in human data (DOI: 10.1073/pnas.1708383114) and dendritic cell populations are very small in the organoids, so this is unlikely to have a major impact regardless. In monocytes/macrophages, some studies suggest that hemozoin biases M1 polarization while other studies suggest the opposite (doi: 10.1128/IAI.01327-06; DOI: 10.1038/s41598-025-30231-x; doi: 10.1002/mbo3.651). We have added to the discussion section that “Future studies should dissect the contribution of different parasite components (including hemozoin, a byproduct of iRBC lysis)” and “test the addition of other parasite stages including merozoites” (lines 443-444).

The authors muse that maybe a lack of changes in antibody responses is that the cultures are not sufficiently long enough but the splenic organoids were cultured for 28 days. They mention there was little to no anti-Plasmodium antibody response but this seems odd given the likely contribution of extrafollicular responses. I would expect to see at least some IgM. The authors only show IgG.

We appreciate this suggestion from the reviewer. In the revision, we now provide IgM data and have included that data in Figure S6E-F. Despite significant background signal in some donors, there were detectable IgM responses (higher for iRBC-stimulated cultures as compared to uRBC-stimulated or unstimulated) in approximately half of the donors. Reponses were generally higher at day 7 as compared to day 14. Although the increase in IgM at Day 7 was not statistically significant, it does suggest that the organoid system could be used to study primary immune responses to Plasmodium parasites (lines 358-360).

Reviewer #2: 1. The details of the organoid cultures and validation of their 3D structure is missing (i.e. images of their structure, staining of their structure by IHC etc) and this is important for the interpretation that these findings are in organoid structures or simply co-cultures of spleen/tonsils with iRBCs?

Although additional imaging of the organoid cultures would be beneficial, we do have confidence in the structure of the organoids. Wagar et al. (2021) performed confocal microscopy of LAIV-stimulated tonsil organoid cultures and observed B and T cell areas in the germinal center with light and dark zone organization and expected distribution of different cell types. By light microscopy, our organoids stimulated with iRBCs appear similar to the LAIV-stimulated organoids. The spleen organoid function is similar to tonsils and thus we expect similar spatial organization; however, optimizing visualization of spleens has unfortunately been challenging.

2. The data that support the claim that Vd2 TCRs clonally expand is not clear enough to interpret. I don't know what TCR chain these figures relate to or it's not clearly in Figure legend. It's well know that the Vd2 TCR repertoire is semi invariant - Vg9 repertoires contain highly shared CDR3 sequences and often exist as presumably clonally expanded populations - however they often pair with different Vd2 TCR sequences. The authors need to show either Vd2 clonotyping or paired sequencing and more convincingly describe how this is clonal selection.

We appreciate this point. To make it clearer that clonal expansion is occurring, we have edited Figure 3C to show where the top 10 TCR sequences fall on the UMAP and have created a supplementary table showing frequencies of unique CDR3 sequences (Supplemental Table 1).

3. How do these "tissue specific" cellular and TCR responses compare to blood, it would ideal to see data on this, but failing this a detailed discussion of if these responses are an extension of observations in blood or lymphoid tissue specific?

We agree that this would be an interesting comparison. Unfortunately, we do not have corresponding blood samples for most of the experiments performed (we did include blood samples in the single-cell experiment for 2/3 donors, but unfortunately it was very poor quality given that it came from a deceased individual). We have added additional discussion comparing our results to what is known in circulating cells of both malaria-naïve and malaria-exposed individuals (see response to Reviewer 1 above).

Reviewer #3: n/a

**Part III – Minor Issues: Editorial and Data Presentation Modifications**
Please use this section for editorial suggestions as well as relatively minor modifications of existing data that would enhance clarity.

Reviewer #1: There are a few reported deficiencies in vaccine responses in Plasmodium infected individuals Figure 5D shows the responses to LAIV is in part for half of the samples present in the unstimulated cells showing active plasma cells. The lack of difference when LAIV is cultured in the presence of iRBCs vs uRBCs leading to the conclusion that there is likely no effect of malaria on vaccine responses should be removed – I really think the organoid system is just not sufficiently developed to make any kind of judgement on this issue based on the data shown.

We agree with the reviewer that our data and do not argue that our data shows that “there is likely no effect of malaria on vaccine responses”. In contrast, we state: “these results do not support the hypothesis that exposure to malaria may impair responses to recall vaccination with influenza; however, this does not exclude the possibility of this relationship at other timepoints or following exposure to other pathogens/vaccines” (lines 368-369), which we feel accurately describes the results. The discussion also acknowledges that limitations of the organoid system “could have influenced our observation that iRBC exposure and γδ T cell depletion do not impact antibody responses to LAIV” (lines 442-443). Given that the majority of the manuscript focuses on responses to malaria, we think these experiments provide an interesting outlook to investigate the secondary impact on vaccine responses even if our data is not conclusive.

Reviewer #2: The materials and methods and the description of the organoid cultures need improvement, and as mentioned more detailed and robust analysis of the TCR repertoires is required to corroborate the claims in this paper.

We have provided detailed descriptions of the organoid cultures in the methods (Lines 475-543). If there are additional details that the reviewer would like us to include, we would be happy to add them. We believe the concern about the TCR repertoires has been addressed in Figure 3C and Supplemental Table 1.

Figure 1 - how do the various frequencies compare to D0 spleen and tonsil samples without culture? Can this be plotted alongside the cultured control conditions.
Figure 1 - Do you have any PBMC cultures to compare these frequencies to?

We have now added D0 frequencies for 7 donors for which we had additional cells available to Figure 1B. Although there were some differences frequencies in some cell types between D0 and D7, these were not significant after multiple comparison correction. We unfortunately do not have PBMC cultures to compare these frequencies to, though we agree that that would be an interesting future experiment.

Reviewer #3: The authors should include the flow cytometry gating strategy for Vδ2⁺ γδ T cells in both the organoid experiments and the Ugandan tonsil samples. In addition, the authors should clarify whether Vδ1 and Vδ3 were analyzed in the organoid system and tonsillar samples, and show the corresponding plots if available. The gating strategies for γδ, Vδ1⁺, and Vδ2⁺ subsets should be plotted and included in the main figures.

Gating strategies for γδ T cells have been moved to main figures (Figure 1F for the naïve organoid experiments and Figure 4A for the Ugandan tonsil samples). Full gating strategies for all cell types in the naïve organoids remain in Figure S1. In the naïve organoids, Vδ1+ and Vδ2+ populations were analyzed. In the Ugandan samples, there was no Vδ1 marker in the panel, so populations analyzed were Vδ2+ and Vδ2-.

Providing a visual schematic of the Vδ2/Vδ1 dynamics in naïve, primary infection, and chronically exposed individuals would contextualize the Press et al. findings within the latest γδ T-cell biology and significantly enhance the manuscript.

Thank you for this suggestion - we have considered it carefully. However, given that we feel that our results in the organoids primarily support what is already known about γδ T cells in the blood following malaria infections (with perhaps the exception of increased Vδ2+ T cell clonal expansion in the organoids compared to blood), we do not feel that a visual schematic would contribute significantly.
